# Acute Ischemic Stroke and Myocardial Infarction in a Patient Diagnosed With *Vibrio cholerae* Infection: A Case Report and a Literature Review

**DOI:** 10.1155/crdi/8899020

**Published:** 2025-11-10

**Authors:** Asiya Aqeel Thakur, Afia Aziz, Ala Osama Elmabrook Hassan, Vazgen Mnatsakanyan, Osman Koc, Ziad Alsehli, Fazilu Rahiman Keelath, Yahia Imam

**Affiliations:** ^1^Department of Medicine, Hamad Medical Corporation, Doha, Qatar; ^2^Neuroscience Institute, Hamad Medical Corporation, Doha, Qatar; ^3^Department of Cardiology and Cardiovascular Surgery, Heart Hospital, Hamad Medical Corporation, Doha, Qatar; ^4^College of Medicine, Qatar University, Doha, Qatar; ^5^Weill Cornell Medicine, Doha, Qatar

**Keywords:** ischemic stroke, ST-elevation myocardial infarction, *Vibrio cholerae*

## Abstract

Cholera is an acute diarrheal disease caused by *Vibrio cholerae* which primarily affects the gastrointestinal system. However, severe dehydration and electrolyte imbalances caused by diarrhea can precipitate systemic complications, including renal, cardiovascular, and cerebrovascular events. This case report describes a rare occurrence of cholera with concomitant myocardial infarction (MI) and ischemic stroke that has not been previously reported in the literature.

## 1. Introduction

Cholera is a contagious disease caused by toxicogenic strains of *Vibrio cholera*. It manifests as a life-threatening diarrheal illness with an estimated mortality of 21,000 to 143,000, as per the CDC. It is known to be transmitted via the fecal-oral route or through the consumption of contaminated water resources from the environment [1–3].

*Vibrio cholerae* is a curved, rod-shaped, Gram-negative bacterium with a single long flagellum for motility. It has around 200 serogroups; however, the disease-causing serogroups responsible for the pandemics are mainly O1 and O139 [4]. The cholera toxin and the toxin coregulated pilus play important roles in the pathophysiology of secretory diarrhea. The toxin coregulated pilus aids in the colonization of the intestines by *Vibrio cholerae*, while cholera toxin induces fluid secretion by a mechanism that causes persistent activation of adenylate cyclase. This activation causes an increase in intracellular cAMP which in human intestinal epithelial cells is responsible for profuse diarrhea and severe fluid loss in cholera [5]. The resulting severe hypovolemic shock caused by extensive fluid losses can be highly fatal, with a case fatality rate surpassing 50% if not managed appropriately with aggressive intravenous fluid hydration [6].

Significant electrolyte imbalances can occur, which include hyponatremia or hypernatremia, hypocalcemia, hypokalemia, hypoglycemia, and acidosis [7, 8]. It can also lead to major complications like renal failure, aspiration pneumonia, chronic enteropathy, and malnutrition in children, and is also uncommonly known to cause fluid accumulation within the intestinal lumen (cholera sicca) [7, 9].

Nevertheless, cholera-related vascular problems such as stroke and myocardial infarction (MI) are exceedingly rare. We conducted a comprehensive literature search using PubMed, Google Scholar, and ScienceDirect, which yielded just one further case report documenting a neonate with cholera-related stroke and meningitis [10]. We found an additional case of cholera-related myocarditis during our literature search [11]. However, we did not come across any reports linking cholera to vascular complications like MI or stroke in adults. Here, we describe an interesting and rare complication of stroke and MI in a patient who came with cholera from India.

## 2. Case Presentation

A 47-year-old Indian male presented to our government hospital in Doha, Qatar, in May 2024, complaining of persistent watery diarrhea, without hematochezia or mucoid content, lasting one day and exceeding over forty episodes. The patient also reported experiencing colicky, nonradiating, paraumbilical abdominal pain, diaphoresis, lethargy, and recurrent nonprojectile vomiting containing food particles. There were no associated symptoms of dysphagia, hematemesis, or melena. He denied any sick contacts but reported consuming food from a local restaurant in India one day before the onset of acute diarrheal illness.

The patient had no significant past medical or surgical history except for previous cataract surgery. He is a nonsmoker with no personal or family history of inflammatory bowel disease or other gastrointestinal disorders. He returned from India the day before his hospital presentation, and no other family members had similar gastrointestinal symptoms during the same period.

Upon arrival at the emergency department, he was afebrile with a normal pulse rate, and a supine blood pressure of 91/54 mmHg. Complete physical examination was otherwise unremarkable. Initial laboratory findings are outlined in Table 1. Upon evaluation, the patient was found to have hypotension, metabolic acidosis with a normal anion gap, and acute kidney injury (AKI), and was subsequently admitted to the medical intensive care unit (ICU). As a result of elevated troponin levels, serial troponins and an echocardiogram were requested. Initial ECG showed normal sinus rhythm without ST-segment elevation or T wave changes. Stool and blood cultures were obtained and treatment with intravenous hydration, sodium bicarbonate tablet administration, and the initiation of intravenous ceftriaxone and metronidazole were commenced for a total of seven days.

**Figure 1:**
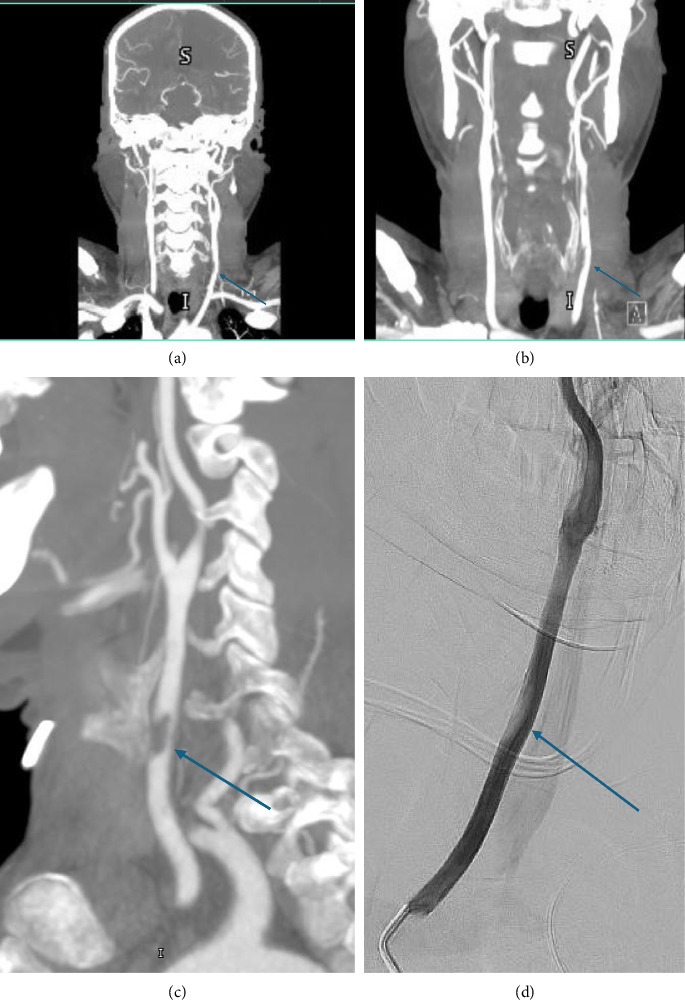
Marked arrows show fresh thrombus at left common carotid artery wall. From left to right (a) and (b) CT angiogram coronal view, (c) CT angiogram sagittal view, and (d) cerebral angiography showing a filling defect indicating a fresh thrombus at left common carotid artery.

A few hours after the admission, the patient exhibited disorientation and incoherent vocalizations upon exiting the restroom. Nursing staff noted labored breathing along with new right-sided hemiplegia, and aphasia. Upon re-assessment, he was drowsy, opening his eyes to painful stimulation, mute, not following commands, with a left gaze preference. He was noted to have flattening of the right nasolabial fold and right-sided dense hemiplegia, with a National Institutes of Health Stroke Scale Score (NIHSS) of 22. Urgent CT head and CT angiogram imaging showed occluded left anterior cerebral artery (ACA) branches distal to the anterior communicating artery (ACOM) and atheromatous changes with significant stenosis in the left common carotid artery (CCA) (Figure 1).

**Figure 2:**
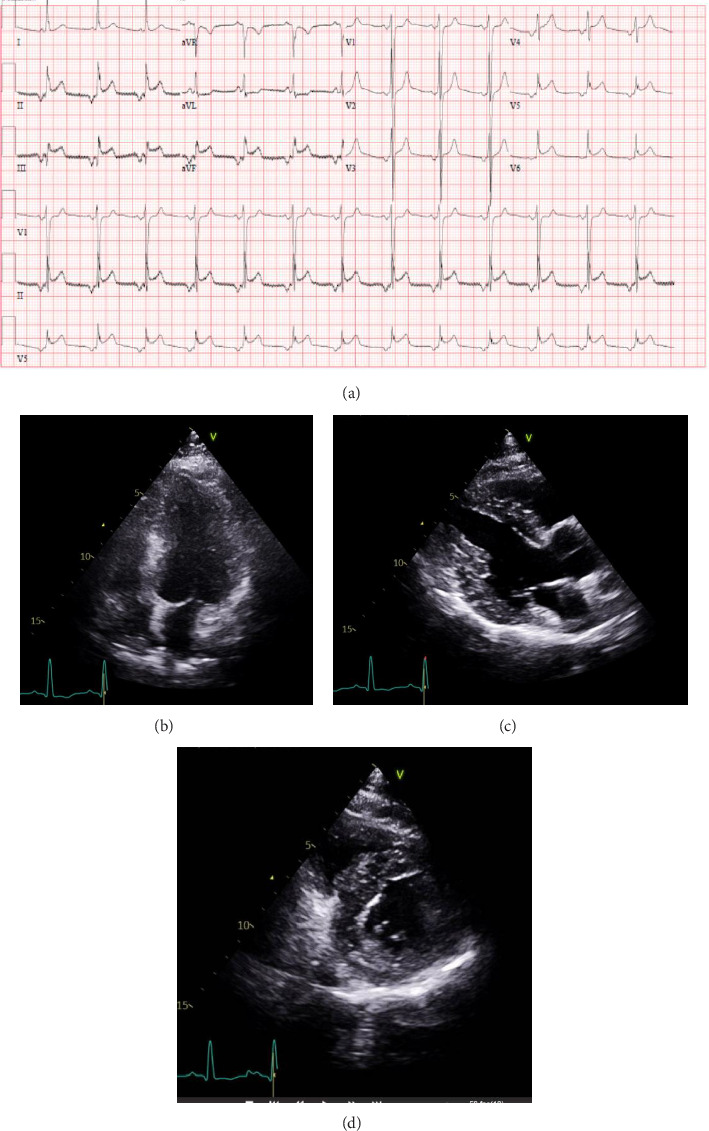
From left to right: (a) ECG of the patient at 25 mm/s, 10 mm/mV, 150 Hz showing junctional tachycardia with inverted P waves and ST elevation in inferior leads II, III, and aVF without clear reciprocal changes and ST elevation in V5, and V6, (b) transthoracic echocardiogram apical four chamber view, (c) transthoracic echocardiogram parasternal long axis view, and (d) transthoracic echocardiogram parasternal short axis view. Transthoracic echocardiogram demonstrated a left ventricle ejection fraction of 61% with no evidence of regional wall motion abnormalities or diastolic dysfunction.

Furthermore, his follow-up ECG showed junctional tachycardia with inverted P waves and ST elevation in inferior leads II, III, and aVF without clear reciprocal changes and ST elevation in V5 and V6 (Figure 2(a)). His troponin levels showed a progressive increase from 21 to 57 to 120 ng/L. The patient denied experiencing chest pain during the initial presentation. However, he was aphasic and disoriented at this stage which hindered the collection of further cardiac history. His echocardiogram demonstrated a left ventricle ejection fraction of 61% with no evidence of regional wall motion abnormalities or diastolic dysfunction (Figures 2(b), 2(c), and 2(d)).

Both the cardiology and neurology stroke teams were involved in the patient's care. The initial assessment by the cardiology team was primary acute coronary syndrome (ACS) leading to ST-segment elevation MI (STEMI), or acute myopericarditis, or prolonged coronary spasm related to metabolic acidemia. However, due to concurrent presence of encephalopathy, hemiplegia, and severe acidosis, they deemed that cardiac intervention would be risky. They advised medical management with dual antiplatelet therapy (DAPT) and heparin infusion if not contraindicated, along with statin and beta-blocker therapy.

**Figure 3:**
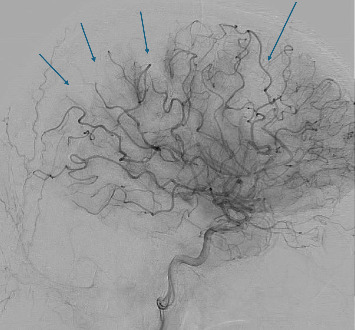
Digital subtraction angiogram (DSA) showing multiple emboli at distal branches of left anterior cerebral artery (ACA)–middle cerebral artery (MCA) territories (arrows).

The stroke team arranged for a potential thrombectomy by the interventional radiology (IR) team, following which the patient was transferred to the neurocritical care unit. The IR team performed selective catheterization of the left CCA. Cerebral angiography showed a fresh thrombus at the common carotid wall (Figure 1(d)). Multiple emboli were observed in the distal left anterior and middle cerebral artery branches (Figure 3). The clot extraction was deemed to be unreachable and risky by the IR team. The stroke team initially advised maintaining the patient on triple therapy (DAPT and heparin infusion) due to the high risk of thrombus embolization, while being cautious of the risk of hemorrhagic transformation and bleeding. Thereafter, the stroke and the critical care team decided to start treatment with a single antiplatelet agent, aspirin, and heparin infusion. Additionally, a follow-up MRI was scheduled, along with a carotid Doppler ultrasound to assess for the presence of stenosis, thrombus, or plaques. Furthermore, echocardiography with contrast was scheduled to rule out the presence of cardiac thrombus in the left ventricle.

**Figure 4:**
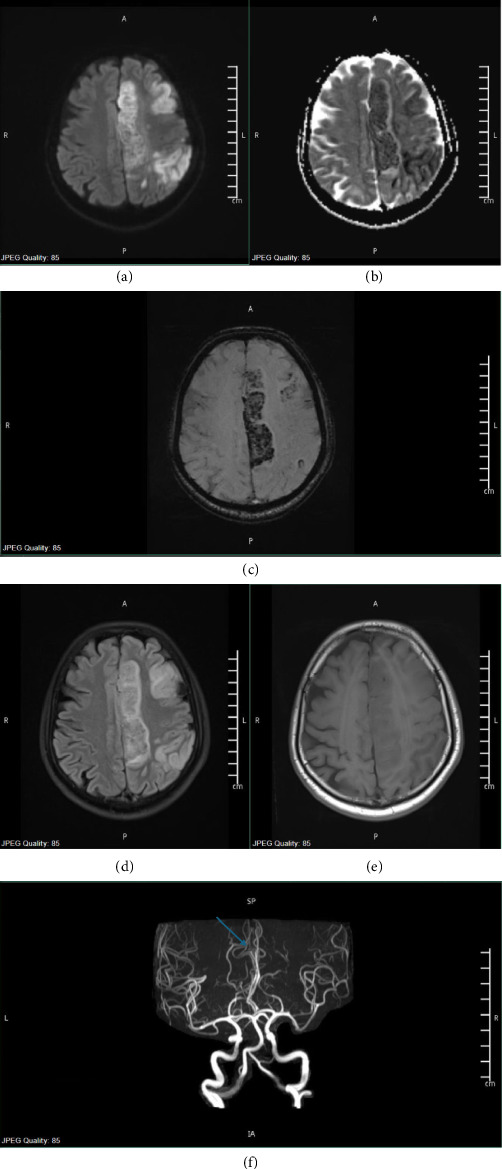
From left to right (a) and (b): This is a magnetic resonance imaging (MRI) axial diffusion-weighted long b value image (DWI) and apparent diffusion coefficient (ADC) map showing infarct in left anterior cerebral artery (ACA)-middle cerebral artery (MCA) territories. (c) This is an axial magnetic resonance imaging (MRI) susceptibility weighted image (SWI) showing extensive blooming artifact in the left parasagittal frontoparietal region consistent with hemorrhagic transformations. (d) T2 FLAIR sequence. (e) T1 sequence-extensive multifocal confluent parenchymal areas of diffusion restriction and T2/fluid attenuated inversion recovery (FLAIR) images showing hyperintense signal intensity in the left fronto-parieto-occipital cortical/subcortical, high left parasagittal frontoparietal regions. (f) 3D-time of flight (TOF) intracranial magnetic response angiography (MRA) showing attenuated caliber mid A2 segment of the left ACA (as compared to the right side) with no definite occlusion; however, there is asymmetric paucity of the distal branches.

On the third day of admission, stool culture confirmed the presence of *Vibrio cholerae*. Infectious Diseases team recommended initiating azithromycin therapy for one day. Follow-up MRI/MRA imaging revealed extensive recent infarctions with hemorrhagic transformation in the left fronto-parieto-occipital region (Figure 4). The stroke team then advised discontinuing heparin infusion and decided to keep the patient on aspirin therapy alone to mitigate the risk of bleeding after noting resolution of the CCA thrombus. They further recommended a follow-up CT head after one week to assess the potential for reintroducing DAPT. The patient's troponin values had peaked at a maximum of 1646 ng/L by the third day of hospital admission, after which a downtrend in levels was observed in the subsequent days.

**Figure 5:**
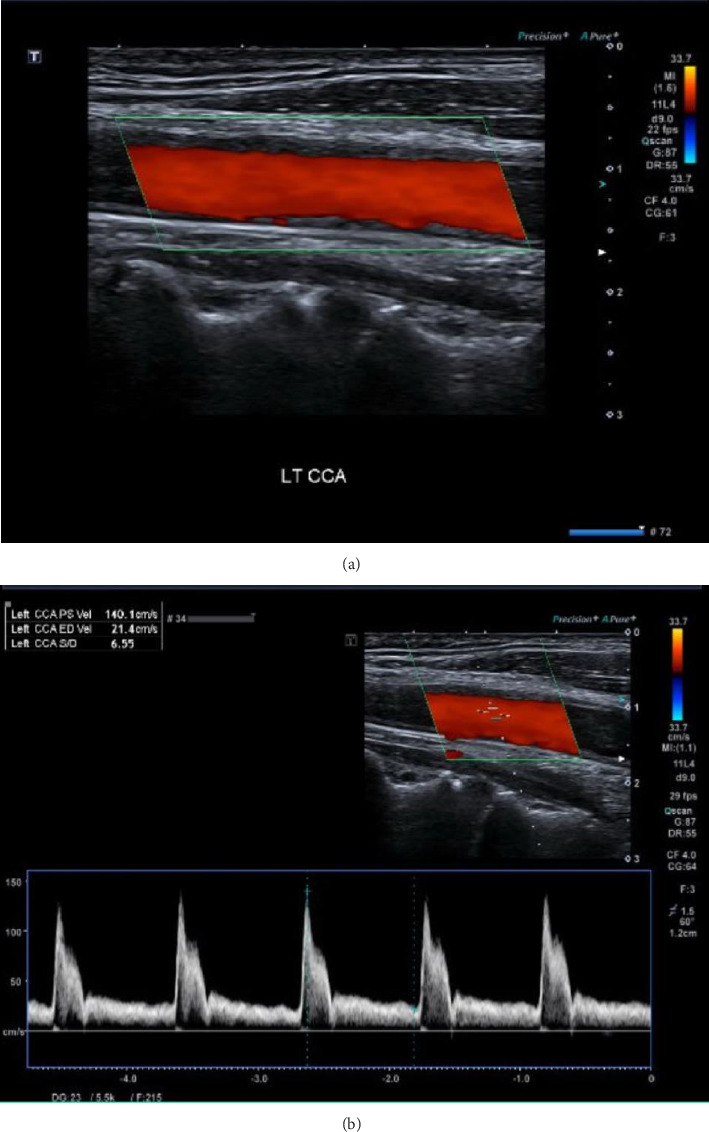
(a) and (b) show follow up carotid Doppler image of the patient after 3 days of anticoagulation showing normal duplex ultrasonography of left common carotid artery without any evidence of focal functional luminal stenosis or flow disturbances.

The patient was transferred to the stroke unit due to stable clinical status without further deterioration. A follow-up CT head performed one week later revealed a re-demonstration of the left fronto-parietal, parasagittal, and cortical recent ischemic infarct along the ACA/MCA territories with hemorrhagic transformation. A mass effect was noted in the form of effacement of the left fronto-parietal cortical sulci with no hydrocephalus or significant midline shift. The carotid Doppler ultrasound findings were unremarkable with no focal functional luminal stenosis or flow disturbances noted bilaterally (Figure 5). Additionally, contrast-enhanced echocardiography did not show the presence of any thrombus in the left ventricle.

**Figure 6:**
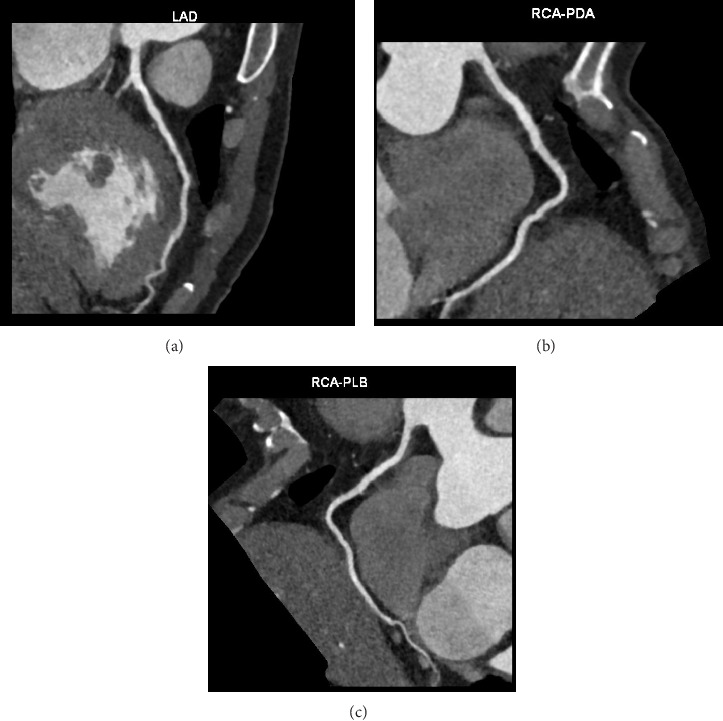
CT cardiac coronary angiography shows no significant coronary artery disease with a calcium score of zero. From left to right: (a) image shows left anterior descending (LAD) artery, (b) image shows right coronary artery-posterior descending artery (RCA-PDA),and (c) image shows right coronary artery-posterolateral branch (RCA-PLB).

In light of these findings, the stroke team had opted to continue with aspirin therapy alone and recommended a follow-up head CT in four weeks. The cardiology team confirmed their decision to maintain the patient on conservative medical treatment for acute inferior ST-elevation MI due to hemorrhagic transformation of acute ischemic stroke and downtrending troponin levels, with plans for outpatient coronary angiography following neurological recovery. During the initial presentation, it was not feasible to do the CT cardiac coronary angiography as the patient was not cooperative due to his neurological status. A month later, CT cardiac coronary angiography was performed; however, it was a suboptimal study due to poor breath-holding effort. No evidence of significant coronary artery disease was noted in this imaging with a calcium score of zero (Figure 6). Considering these results, the cardiology team finally concluded that the most probable diagnosis in this patient was transient coronary emboli due to dehydration or infection. Myocarditis could have been another differential; however, it was thought to be the least probable cause in our patient's case.

The patient is currently showing clinical improvement despite being aphasic with dense right-sided hemiplegia. His diarrhea has resolved, and his kidney function has fully recovered. He has completed the antibiotic course for the treatment of cholera and is undergoing physical rehabilitation with physical and occupational therapy.

## 3. Discussion

Various infections, including bacterial, parasitic, fungal, and viral etiologies such as tuberculous meningitis, neurocysticercosis, mucormycosis, and HIV vasculopathy, have been directly associated with causing stroke [12]. Recent evidence has identified SARS-CoV-2, the virus responsible for the COVID-19 pandemic, as a new causative agent of stroke [13, 14]. The presence of unusual organisms in nonendemic regions is widely increasing due to travel. Stroke has been frequently reported in travelers arriving at or transitioning through busy international hubs [15]. However, our literature search performed on databases like Google Scholar, ScienceDirect, and PubMed in June 2024 revealed that there have been no reported cases linking vascular complications like ischemic stroke and MI to *Vibrio cholerae* infection. This case report aims to elucidate the possible mechanisms that may have contributed to the development of this complication.

Historically, cholera has been known to cause fatal diarrheal illness before the advent of modern treatments such as intravenous fluid resuscitation, oral rehydrating solutions, and oral cholera vaccines. This waterborne disease continues to pose a significant global health threat, with an estimated 1.3 to 4.0 million cases occurring annually [16]. Cholera infection can lead to rapid fluid losses and severe electrolyte and acid-base imbalances, potentially leading to severe hypovolemic shock if not treated promptly and aggressively with fluid resuscitation. Complications associated with cholera include renal failure, aspiration pneumonia, chronic enteropathy, malnutrition in children, and also, uncommonly, fluid accumulation within the intestinal lumen (cholera sicca) [7, 9]. Our case is extremely unique in that our patient experienced an incredibly rare complication of ST-elevation MI and acute ischemic stroke, which has not been previously reported in the literature. This alarming development highlights the complexities of cholera infection and underscores the need for further understanding and research into its potential impacts on the cardiovascular system.

Our patient exhibited signs consistent with severe dehydration. We have noted a significant elevation in red blood cell and white blood cell count in our patient upon presentation. The concentration of blood cellular components (red cells, leukocytes, or platelets) determines the viscosity of the blood, and the viscosity quantifies how resistant a fluid is to deformation. An increase in any of these components could result in hyperviscosity of the whole blood. A three-fold increase in viscosity could be noted when the hematocrit ranges between 50% and 80% [17, 18]. An increase in the red blood cell count or the hematocrit can be particularly observed in dehydration where there is shrunken plasma volume. Loss of water from the plasma compartment also increases the protein concentration which can lead to an elevation in the viscous state [17]. Elevated total protein was also observed in the laboratory findings of our patient.

Hyperviscosity accounts for fatal thromboembolic complications in disease states like polycythemia vera. Data from an Italian retrospective cohort study, where 1213 patients with polycythemia vera were followed for 20 years, showed that arterial thrombosis accounted for about two-thirds of thrombotic events, out of which MI and ischemic stroke accounted for the vast majority of fatal thrombotic complications [19]. Thus, in our patient, we hypothesized that relative polycythemia due to volume contraction and severe dehydration led to a hyperviscous state which resulted in arterial thrombotic complications, like that of polycythemia vera.

Our patient likely developed embolic complications resulting from hyperviscosity similar to that seen in COVID-19 pandemic from floating carotid thrombus [13]. This conclusion can be drawn from the fact that a large fresh thrombus was noted in the left CCA in cerebral angiography with showered emboli occluding distal left anterior and middle cerebral arteries. Carotid Doppler ultrasound done in the following days did not show any evidence of occlusion or thrombus in the carotid arteries indicating that the thrombus had already embolized into the distal circulation. Similarly, no evidence of thrombosis was noted in the CT cardiac coronary angiography of our patient which made us reflect that ST-elevation MI could also have been due to a transient coronary emboli in normal coronary arteries. MI with nonobstructive coronary arteries (MINOCA), a clinical entity that is thought to occur secondary to coronary microvascular dysfunction or coronary artery spasm or plaque disruption, may have been the likely cause in our patient [20].

Severe metabolic acidosis in our patient may also have contributed to increased risk of cardiovascular events. A large retrospective cohort study done in patients with chronic kidney disease demonstrated that the incidence and risk of MI and stroke was higher in patients with metabolic acidosis as compared to those with normal serum bicarbonate level. The mechanism by which this occurs is still uncertain; however, increased inflammation leading to endothelial dysfunction could be implicated in metabolic acidosis [21].

This case has prompted us to consider if the cholera toxin has any direct role to play in the development of myocardial ischemia. Several animal studies conducted with cholera toxin in the past have demonstrated the cyclic AMP (adenosine 3′,5′-cyclic monophosphate)-dependent positive inotropic effect of cholera toxin [22, 23]. However, it is yet to be determined if cholera toxin can directly induce ischemia and necrosis, and this can be a scope for future studies. There has been one case report published by Leon et al. in 1997 where they found a direct association linking a patient with cholera infection to myocarditis. This was confirmed by myocardial tissue biopsy, which showed a positive polymerase chain reaction and antitoxin cholera antibodies in immunohistochemical testing. Coronary angiography done in this patient revealed normal coronaries with no evidence of MI [11].

One of the limitations present in our case is that we were unable to obtain a coronary angiography in our patient as it was deemed to be a high risk, given the development of large acute ischemic stroke with hemorrhagic transformation. We were, hence, unable to determine if the patient's ST-elevation MI was a result of microvascular or macrovascular occlusion during the time of acute insult. Nonetheless, a normal CT cardiac coronary angiography led us to believe that transient emboli may have been the cause of infarction.

Another limitation noted in our patient's case is that we did not have coagulation parameters and fibrinogen levels at initial presentation upon hospital admission to determine if his development of stroke and MI could have been secondary to coagulopathy conditions like disseminated intravascular coagulopathy.

Despite the above-mentioned limitations, to our knowledge, ours is the first reported case linking vascular complications like MI and stroke to *Vibrio cholerae* diarrheal illness.

## 4. Conclusion

This case has shed light on the need to gather more evidence regarding thrombotic complications in patients diagnosed with cholera infection. Further studies, case reports, case series, and observational studies are needed to determine the incidence and prevalence of vascular complications due to severe dehydration in cholera. Studies also need to be conducted to further understand the mechanism and pathophysiology of vascular complications in cholera.

## Figures and Tables

**Table 1:** Initial laboratory findings of our patient.

Details	Values with units	Normal range
WBC	**19.9 × **10^3^**/μL (high)**	(4.0–10.0) × 10^3^/μL
RBC	**6.5 × **10^6^**/μL (high)**	(4.5–5.5) × 10^6^/μL
Hemoglobin	**19.2 gm/dL (high)**	13.0–17.0 gm/dL
Platelet	**421 × **10^3^**/μL (high)**	(150.0–410.0) × 10^3^/μL
Absolute neutrophil count	**17.4 × **10^3^**/μL (high)**	(2.0–7.0) × 10^3^/μL
Cholesterol	7.8 mmol/L	
Triglyceride	3.2 mmol/L	
HDL	1.2 mmol/L	
LDL–calculated	5.2 mmol/L	
Beta-hydroxybutyrate	0.18 mmol/L	0.03–3.0 mmol/L
C-reactive protein	**7.3 mg/L (high)**	0.0–5.0 mg/L
Lactic acid	2.0 mmol/L	0.5–2.2 mmol/L
Procalcitonin	0.13 ng/mL	
Urea	3.9 mmol/L	2.5–7.8 mmol/L
Creatinine	**150 μmol/L (high)**	62–106 μmol/L
Sodium	136 mmol/L	133–146 mmol/L
Potassium	**5.6 mmol/L (high)**	3.5–5.3 mmol/L
Chloride	107 mmol/L	95–108 mmol/L
Bicarbonate	**14 mmol/L (low)**	22–29 mmol/L
Total bilirubin	11 μmol/L	0–21 μmol/L
Alkaline phosphatase	**140 U/L (high)**	40–129 U/L
ALT	**89 U/L (high)**	0–41 U/L
AST	**46 U/L (high)**	0–40 U/L
Total protein	**98 gm/L (high)**	60–80 gm/L
Albumin	**52 gm/L (high)**	35–50 gm/L
Troponin-T HS	**21 ng/L (high)**	3–15 ng/L
Glucose random	12.9 mmol/L	
pH venous POC	**7.02 (low)**	7.35–7.45

*Note:* The initial labs revealed acute kidney injury and normal anion gap metabolic acidosis, and bold values indicate significant hemoconcentration suggestive of severe dehydration.
